# Comment to: Planned iliohypogastric neurectomy for prevention of chronic pain after inguinal hernia repair

**DOI:** 10.1007/s10029-025-03412-w

**Published:** 2025-07-09

**Authors:** Christoph Paasch, R. Fortelny

**Affiliations:** 1 Department of General and Visceral Surgery, University Hospital Brandenburg an der Havel, Hochstraße 29, 14770 Brandenburg an der Havel, Germany; 2 Medical faculty, Head of General Surgery Sigmund Freud Private University Vienna, Vienna, Austria

**Dear Editor**,

We recently read the article *‘Planned iliohypogastric neurectomy for prevention of chronic pain after inguinal hernia repair’* with great interest. The manuscript was published in 2025 [1]. The authors clearly invested significant effort in conducting this randomised clinical trial (RCT) and successfully recruited 1,375 patients. We sincerely appreciate their work and commitment to advancing the field.

However, we believe that several important methodological aspects were not sufficiently addressed by the authors.

First, the study was not registered in a clinical trial registry. Prospective registration is a mandatory requirement for RCTs and essential for transparency and reproducibility. The authors present a power calculation setting the required sample size at 252 participants per group. However, no supporting literature is cited. Ultimately, 534 patients were included in Group 1 and 594 in Group 2. This discrepancy in sample size, combined with the lack of a registered protocol, limits the reproducibility of the trial and does not align with current best practices for clinical research.

Second, the primary endpoint — postoperative pain following open hernia repair with mesh insertion — was assessed using a non-validated pain score. In RCTs evaluating pain outcomes, validated instruments such as the Visual Analogue Scale (VAS) or Numeric Rating Scale (NRS) should be used to allow meaningful comparison with the existing literature and to ensure methodological rigor.

Third, according to the description provided in the article, patients underwent open inguinal hernia repair with mesh insertion (Lichtenstein repair? ). In our view, and in accordance with Amid’s updated description of the Lichtenstein procedure [2, 3], a non-absorbable running suture should be used to fix the mesh to the inguinal ligament. This appears to contrast with the technique reported in the present study.

We do not wish to discourage the authors in any way and appreciate their contribution to the field. However, we feel it is our responsibility to point out these major methodological issues, which should be taken into account when interpreting the study’s results.

## Electronic supplementary material

Below is the link to the electronic supplementary material.

Supplementary Material 1

Supplementary Material 2
